# The COVID-19 pandemic: the benefits and challenges it presents for medical education in Africa

**DOI:** 10.11604/pamj.2021.40.42.28489

**Published:** 2021-09-16

**Authors:** Anthonio Oladele Adefuye, Henry Ademola Adeola, Jamiu Busari

**Affiliations:** 1 Division of Health Sciences Education, Office of the Dean, Faculty of Health Sciences, University of the Free State, P.O. Box 339, Bloemfontein 9300, South Africa,; 2 Department of Dermatology, Faculty of Health Sciences, University of Cape Town, Cape Town, South Africa,; 3 Educational Development and Research Department, Faculty of Health, Medicine and Life Sciences, Maastricht University, Maastricht, The Netherlands

**Keywords:** COVID-19, medical education, sub-Saharan Africa

## Abstract

The coronavirus disease (COVID-19) has impacted many facets of everyday daily life, resulting in far-reaching consequences on social interaction, regional and global economies, and healthcare delivery systems. Numerous reports have commented on the impact of the COVID-19 pandemic on medical education in various world regions. However, we know little about the influence of the pandemic on medical education in Africa. Here, we discuss the potential impact of COVID-19 on teaching and learning in undergraduate medical education in sub-Saharan Africa, illustrating some of the unexpected benefits and challenges the pandemic presents for medical education in sub-Saharan Africa.

## Introduction

Since its onset, the coronavirus disease (COVID-19) has impacted many facets of everyday life, resulting in far-reaching consequences on social interaction, regional and global economies, and healthcare delivery systems. Similarly, numerous reports have commented on the impact of the COVID-19 pandemic on medical education in various world regions. Documented reports include the transition to distance online education by medical faculties in Europe [1]; conduction of the first-ever online exam for final years by Imperial College London [2]; adoption of an open-book examination approach by some medical schools in the United Kingdom[1]; implementation of an online pre-clerkship curriculum by medical education faculties in the United States of America [3]; and the use of simulations and virtual learning platforms by medical schools in Asia [4]. It is still unclear what the influence of the COVID-19 pandemic is on medical education in Africa. Hence, we explore the potential impact of COVID-19 on teaching and learning in undergraduate medical education in Sub-Saharan Africa, highlighting some of the benefits and challenges the pandemic it presents for medical education in sub-Saharan Africa.

### Undergraduate medical education in sub-Saharan Africa before the onset of the COVID-19 pandemic

According to the latest World Directory of Medical Schools (updated November 2020), Africa has 154 operational medical schools spread across 54 nations [5]. The education and training in these institutions are regulated by the respective statutory body established in each country. For example, the Medical and Dental Council of Nigeria (MDCN) [6] and the Health Professions Council of South Africa (HPCSA) [7]. Before the onset of the COVID-19 pandemic, some medical schools in sub-Saharan Africa had begun implementing new curricula and different pedagogical formats to improve medical education quality in their institutions. Notable examples include the use of problem-based learning and interactive team-based learning as a pedagogy [8], developing competency-based medical education curriculum [9], and integration of traditional, complementary, and alternative medicine into the medical curriculum [10]. Still, colonial-era conventional medical curricula, with little or no modification, continue to be used in many medical institutions to educate trainees. In most of these schools, the undergraduate medical curriculum is characteristically divided into pre-clinical and clinical training with minimal integration between the two, and didactic lectures and traditional instructor-centered strategies being the predominant forms of teaching [11]. Textbooks and lecture notes remain a major teaching resource due to challenges of access to online e-learning opportunities. Furthermore, curricular appraisal and assessment of trainees remain unreliable and subjective [12]. Other challenges to the quality of medical education in sub-Saharan Africa include the perennial problems of insufficient governmental funding, limited infrastructure, and poor faculty development, which continue to plague many African medical institutions. Like many longstanding and vague societal issues such as systemic discrimination, inequity, and poor access to health care, the advent of COVID-19 has exacerbated the challenges and woes of medical education in Africa with many governments across the continent backtracking on their planned increases in education spending in 2020 [13].

### Benefits presented by the ongoing COVID-19 pandemic for medical education in Africa

While the advent of the COVID-19 pandemic has invoked unprecedented economic hardship across the African region, education and particularly medical education, seem to have benefited from this crisis. If looked at closely, the COVID-19 pandemic seems to have paradoxically presented stakeholders with ample opportunities to enhance teaching and learning in undergraduate medical education. There has been a noticeable increase in government spending on technology-based pedagogy [14] and an upsurge in incorporating e-learning within institutions [15]. Furthermore, the number of educators with e-teaching skills has increased, all of which can be ascribed to the COVID-19 pandemic. To further illustrate these “unexpected” benefits of the COVID-19 for medical education in sub-Saharan Africa, we provide an overview of the different arguments for this below:

**Technology-based pedagogy:** the transformation in medical education over the last decades has shown a shift from the traditional face-to-face (Teacher-centered) model of teaching to newer student-centered and competency-based approaches to teaching and learning in undergraduate medical education. A significant change has been the emergence of technology-based pedagogy (E-learning) as a modality. Similarly, in Africa, there is a growing demand for products and services for e-learning; however, due to the absence and/or inadequate coverage of broadband internet and technology infrastructure, the uptake of e-learning as a pedagogy in most parts of the continent is hindered [16]. Hence, teaching in most undergraduate medical curricula in the continent is still via the traditional face-to-face pedagogy with little or no infusion of e-learning modalities.

For most students in the continent, academic institution closures during the COVID-19 pandemic have meant access to little or no academic support or training and delay in the early transition of final year medical trainees into practice. To mitigate the effect of COVID-19 in the education sector, important educational stakeholders and governments across the continent began to invest massively in e-learning. Notable examples of such investment include; establishment of an online platform (Ecole-ci.online - www.ecoleweb.mysonec.com and EducTV Facebook page) by the Ivorian Ministry of Education, Technical and Professional Training via the “*École fermée, mais cahiers ouverts (Closed school but open notebooks)*” initiative [17]; creation of Online Resource Platform by the Senegalese Ministry of Education [18]; and the creation of online resources to support learning at home during the lockdown by the Department of Basic Education in South Africa [19]. Also, many Universities across the continent have developed their online learning management systems [20]. Besides offering laptops, some institutions signed contracts with mobile communication networks to provide their students with free data access to online teaching and learning resources. E-learning has been credited for its advantages, such as enhanced knowledge and skills in undergraduate students compared to the traditional educational methods [21]. Furthermore, it is known that technology-based education enhances medical trainee growth, empowers innovative scientific leaders, stimulates self-reflection, and helps in assimilating relevant information for informed decision-making [22]. It is plausible that the massive investment in technology-based pedagogy, precipitated by the advent of the Covid-19 pandemic, will engender similar attributes in medical graduates across the African continent.

**Innovative ways of teaching:** prior and emerging studies on students' experience on e-learning during the pandemic reports that students become more interactive and engage more with learning course materials when familiar platforms, such as social media sites, are used for online teaching and learning when compared to the learning management system imposed by the University [23]. This meant that medical educators needed to develop innovative ways of teaching in the online environment. Lectures have rapidly been adapted for delivery online, using various content and learning management systems and social media platforms such as Zoom, Skype, Microsoft Teams, WhatsApp, Twitter, Facebook, Pinterest, Snapchat, YouTube, and others [24]. Calls for the customization of learning management systems to meet students' needs and preferences, i.e., linking learning management systems to social media platforms, has been promulgated. The integration of social media platforms into online teaching could lead to improved students' performance by enhancing students' interaction and engagement with learning materials. Uptake of these innovative ways of online teaching could potentially increase educators' effectiveness and efficiency.

**Skilled educators and learners:** due to their inept information technology (IT) skills and the inefficient use of online teaching and learning platforms, most educators and learners' in Africa lack the skills to use e-learning media and learning management systems effectively [25]. The sudden Covid-19-mediated transition from face-to-face teaching and learning to the online teaching and learning platform requires that the users (i.e., educators and learners) of this medium possess the skills and the technological know-how to best use the platform. The pandemic's onset has also brought in its wake an increased awareness and sensitization about e-learning among medical trainees and their educators in the continent. Institutions across the continent have initiated programmes such as the “*Facilitating Online*” a registered short course at the Centre for Innovation in Learning and Teaching, University of Cape Town [26]. Similarly, platforms to train educators and learners about various aspects of online teaching and learning has been created by most institutions in the continent. This will engender the birth of a new generation of medical educators and learners with apt I.T. skill for online teaching and learning.

**Medical faculties and learners:** in low and middle-income countries (LMICs), e-learning in medical education has been reported to confer numerous benefits to learners while simultaneously enhancing faculty effectiveness and efficiency [27]. Some of the benefits of e-learning for students (especially in LMICs) include the easy and increased accessibility to information and the enhanced continuous interaction between students and educators outside the classroom setting. Also, innovations like virtual reality and simulations can effectively increase knowledge, performance skills, and team communication through practical case-based learning sessions [22]. E-learning also encourages students to take responsibility for their learning, building their self-knowledge and self-confidence. E-learning can be used to address faculty shortages and supplement faculty instruction [28] and support learning outside the classroom. Finally, with the aid of mobile technologies, e-learning can be used to enhance faculty effectiveness and efficiency through the implementation of learning in remote and rural areas. It is logical to assume that the exposure of medical faculties and learners in the African continent to e-learning during the COVID-19 pandemic will confer similar benefits.

### Challenges faced by medical education in Africa during the ongoing COVID-19 pandemic

While it is very evident that the emergence of the COVID-19 pandemic has improved the acceptance and use of remote and innovative teaching and learning methodologies in many African countries, it is not without saying that this new normal does not come with numerous challenges. Some of these challenges include:

**Limited infrastructure to accommodate e-learning:** besides impeding factors such as social structure, cultural acceptance, and financial capacity, the lack of adequate infrastructure has been identified as a significant barrier to implementing e-learning in most sub-Saharan Africa countries. For example, access to computers is limited due to affordability, as most workers' average income on the continent is low. On the other hand, access to the internet is restricted, as the cost of accessing the internet in most countries on the continent is still on the high side. The supply of electricity required for seamless online educational engagement of learners is also inconsistent. According to a World Bank report, only 47.7% of the population living in sub-Saharan Africa had access to electricity in 2018 [29]. It was against this pre-existing backdrop that the COVID-19 pandemic arrived in Africa. Thus incapacitating virtual/online clinical and didactic training of medical students, and also restricting access of trainers to students.

**Educators:** being computer-literate and possessing the relevant technical computer/I.T. skills is a crucial factor necessary for the adoption of technology in teaching. Similarly, having confidence in one's skills and ability to use e-learning contributes significantly to the uptake of technology in teaching. This is not so for most educators in sub-Saharan Africa, as an ample number of them still lack skills in the use of e-learning in teaching and learning. The advent of the COVID-19 pandemic thus presents an enormous challenge for most of these educators, as they may find it challenging to develop e-content and employ relevant technologies to teaching.

**Learners:** students are expected to be self-motivated and committed for active learning to occur while engaging in e-learning during the pandemic. However, these expectations may be challenged by poor, inadequate institutional support and, thus, finding it challenging to engage the e-learning platform for active learning. Similarly, students from poor backgrounds may find it difficult to access devices, data, and the Internet because of the high cost and poor coverage in their locality.

**Assessment:** periodic evaluation of students is an essential aspect of most learning and teaching experience. While various online assessment methods have been established for testing and examining students, the uptake and routine use of such e-learning tools are deficient in Africa. Some medical colleges across the continent still indulge in unreliable and subjective methods of assessment, such as viva voce examination and long case/short case clinical presentations [12]. School closures due to the COVID-19 pandemic also interferes with the process of assessment in some of these medical colleges. The inaccessibility to Internet, irregular and frequent interrupted power supply experienced in most part of the continent also poses a significant challenge for online assessment. Furthermore, developing online content for online assessment may be a challenge for educators who are not trained in requisite I.T. skills.

**Financial:** finally, the pre-existing severe financial problems, coupled with the fact that the typical western lenders are also affected by the COVID-19 pandemic, puts an additional layer of strain on successful funding of e-learning programs in Africa [13].

## Conclusion

Even though the number of COVID-19 cases and fatalities are relatively low in Africa, the overwhelming poverty and the paucity of educational, healthcare, and technological infrastructure is significant. These deficiencies can strain the continent's resources and precipitate an educational, healthcare, and socioeconomic crisis. The shortcomings and inequities that the COVID-19 pandemic has laid bare to the world, and in this case, medical education, demands that more advanced e-learning resources (in line with the 4^th^ industrial revolution) should be provided across Africa. Furthermore, policymakers are encouraged to cushion the costs needed for sustainable development of information technology infrastructure within the academic medical school settings and across most universities. Table 1 summarizes some of the challenges posed by the COVID-19 pandemic for medical education in sub-Saharan Africa and possible solutions [30].

**Table 1:** some of the challenges posed by the COVID-19 pandemic for medical education in sub-Saharan Africa

Challenges	Possible solution
**Limited infrastructure to accommodate e-learning**	Stakeholders and government across the continent should pursue a holistic and evidence-based model to inform decisions about investments in infrastructure to accommodate for e-learning. The following proposed steps can be followed; 1) Data collection and analysis - i.e. types of infrastructural needs. 2) Identify infrastructural challenges. 3) Develop set of criteria to prioritize investment. 4) Monitoring - i.e. application of criteria in practice and allocation of fund for infrastructure development.
**Inability of educators to use e-learning in teaching and learning**	Faculties should be encouraged to invest time and money into continuous training of educators on teaching with technology. This could be in the form of short online courses, continuous training workshops and forming an online community of practice to facilitate peer-assisted learning.
**Inadequate institutional support for student to cope with e-learning and teaching**	Faculties and institutions should develop flexible platforms that will allow learners continuous engagement with the institution/educators, enhance student´s connectivity to one another (encourage peer-assisted learning), and support student to succeed in their studies.
**Students´ inability to afford devices for technology-based learning**	Efforts should be made by stake holders in medical education to develop a more inclusive online education system by ensuring that all learners benefit from new technologies. Grant proposals can be written to purchase used and inexpensive devices; solicit for donation of new or used devices through campaigns; local businesses-school partnership or agreement can be entered into for funding; and school can develop flexible platforms that can be accessed on inexpensive devices such as mobile phones etc.
**Difficulty in scheduling face-to-face clinical assessment**	Face-to-face clinical assessment can be substituted with open book exam and a virtually proctored shelf exam [30]
